# Prevalence and risk factors of physical or sexual intimate violence perpetration amongst men in four districts in the central region of Ghana: Baseline findings from a cluster randomised controlled trial

**DOI:** 10.1371/journal.pone.0191663

**Published:** 2018-03-09

**Authors:** Esnat D. Chirwa, Yandisa Sikweyiya, Adolphina Addoley Addo-Lartey, Deda Ogum Alangea, Dorcas Coker-Appiah, Richard M. K. Adanu, Rachel Jewkes

**Affiliations:** 1 Gender and Health Research Unit, South African Medical Research Council, Pretoria, South Africa; 2 Department of Epidemiology and Disease Control, School of Public Health, University of Ghana, Accra, Ghana; 3 Department of Population, Family and Reproductive Health, School of Public Health, University of Ghana, Accra, Ghana; 4 Gender Studies and Human Rights Documentation Centre, Accra, Ghana; University of Westminster, UNITED KINGDOM

## Abstract

**Background:**

Evidence-based interventions are essential in the prevention of violence against women (VAW). An understanding of risk factors for male perpetration of VAW using population-based research is crucial for developing such interventions. This study is a baseline assessment of a two-arm unmatched cluster randomised controlled trial (C-RCT), set up to assess the impact of a Rural Response System (RRS) intervention for preventing violence against women and girls in Ghana. This study aims at assessing past year prevalence and risk factors for sexual or physical intimate partner violence (IPV) perpetration among men.

**Methods:**

The population-based survey involved 2126 men aged 18 and above living in selected communities in 4 districts in the central region of Ghana. Logistic regression techniques were used to determine risk factors for sexual or physical IPV perpetration. All models adjusted for age of respondent and took into account the study design.

**Results:**

Half of the men had perpetrated at least one form of violence against their intimate partners in their lifetime while 41% had perpetrated sexual or physical IPV. Majority (93%) of the men had been in relationships in the 12 months preceding the survey, and of these, 23% had perpetrated sexual or physical IPV. Childhood factors associated with sexual or physical IPV included witnessing abuse of mother (aOR:1.40(1.06–1.86)), and neglect (aOR:1.81(1.30–2.50)). Other major risk factors for IPV perpetration were: having multiple partners (aOR:1.76(1.36–2.26)), (involvement in transactional sex (aOR:1.76(1.36–2.26)), substance use (aOR:1.74(1.25–2.43)) and gender inequitable attitudes (aOR:0.94(0.91–0.97)).

**Conclusion:**

Childhood violence experience and witnessing, risky behaviour (multiple partners, transactional sex, substance use) and gender inequitable attitudes are major risk factors for sexual or physical IPV perpetration. Perpetration of sexual or physical IPV tend to co-occur with non-partner violence and emotional IPV perpetration. Interventions targeting these factors are critical in reducing IPV.

## Introduction

Intimate partner violence (IPV), which refers to aggressive or coercive behaviours among marital, dating or cohabiting partners, remains a global public health concern due to its adverse health consequences to the victims, which are often women [1]. Strong empirical evidence show that IPV constitutes the largest form of violence experience by women, in that women are more likely to be sexually or physically abused by their partners than by non-partners [2–5]. IPV impacts women’s physical well-being, their sexual and reproductive health as well as their mental health [6–10]. Lifetime prevalence of physical or sexual IPV experience amongst women in low-and middle-income countries (LMIC) ranges from 15%-71% globally [3, 11, 12].

Prevalence estimates for women’s IPV experience in Ghana is high. The Ghana Demographic Health Survey 2008 (GDHS) showed that two in five women had experienced either emotional, physical or sexual IPV, and one in five women had experienced physical IPV in their lifetime. The same survey found that one in five women had experienced sexual or physical IPV in the 12 months preceding the survey [5]. Another study conducted in Ghana by the UN Women found similar results on sexual or physical IPV experience by women [12].

Studies amongst men have shown some consistent risk and protective factors for IPV perpetration. Prominent risk factors for IPV perpetration by men include childhood experience of violence (physical or sexual abuse as a child) or exposure to violence (e.g. witnessing abuse of mother at the hands of father or boyfriend) and having permissive attitudes towards violence against women (VAW), while having gender equitable attitudes has been found to be protective against IPV perpetration [4, 11, 13–17].

Substance abuse and mental health issues have also been found to be key risk factors of IPV perpetration by men. Studies have found cumulative risk of depression and post-traumatic stress disorder symptoms and substance abuse to be associated with intimate partner violence perpetration [18–20]. Studies of the relationship between poverty or education (couple relative educational level, financial disparity, employment and poverty indices) and IPV has produced varying results in both strength and direction of association [21–23].

Most LMIC, including Ghana, have patriarchal sociocultural values that condone abuse of women’s rights, and have attitudes that make IPV against women acceptable and culturally normal and reinforcing traditional symbolic structures of male dominance and control over women [13, 21, 24–26]. Dominant attitudes in Ghana are shaped by societal beliefs and norms about traditional gender roles, and views that women’s behaviours justify men’s violent response [24, 27–31]. These are further perpetuated by strong perceptions that violence that occurs in a home is a private/family issue [24]. Furthermore, hegemonic masculinity emphasises men’s control and power over women, substance use, risky behaviour and use of violence [26]. Other factors linked with violence perpetration/victimisation in Ghana include economic and legal factors. Poor economic empowerment of women and social norms that condone abuse of women’s rights are some of the factors that exacerbate abuse of women [4].

In Ghana, despite legislation and advocacy work to reduce the levels of IPV victimisation/perpetration, the effects of such interventions have been inadequate due to limited inclusion of men in such work. Furthermore, most interventions and studies in Ghana have concentrated on women and not included men. The 1998 study by the Gender Studies and Human Rights Documentation Centre (Gender Centre) recommended that the responsibility for men’s perpetration of violence against women and children needs to be shifted to society as a whole rather than put it solely on women [24]. The study also recommended the inclusion of men in all campaigns or interventions aimed at reducing (VAW) [24]. The high degree of tolerance of VAW in the Ghanaian society and the hierarchical and inequitable social structures of culture, religion, and patriarchal family are central in sustaining VAW [24]. The authors argue for transformative interventions that promote gender equitable social norms and attitudes and that discourage tolerance of VAW in society [24]. Other authors have argued that due to entrenched patriarchal norms of male superiority coupled with low level of women empowerment, reducing VAW requires sustained interventions that include men and other stakeholders in the society [13, 14, 32]. The Stepping Stones intervention evaluation in South Africa highlighted the impact of jointly targeted intervention in reducing violence and risky behaviours associated with violence perpetration[33].

This paper examines prevalence of sexual or physical IPV perpetration among men and its associated risk or protective factors in central region of Ghana. It is part of a larger intervention study aimed at promoting gender equitable social norms and attitudes in communities, with the overall aim of reducing violence against women. A number of studies have advanced for interventions that promote gender equitable attitudes among men. These studies have also advanced for interventions that promote positive parenting, and that address normalisation of violence against women and children.

## Methods

The data reported in this paper were drawn from a baseline survey of an unmatched cluster randomised controlled trial evaluating the Rural Response System (RRS) intervention to reduce VAW in Central Region of Ghana. The RRS intervention is focused on both men and women, however for the purpose of this paper only baseline data collected from men is presented.

The baseline survey was done in four districts located in the Central Region of Ghana. These districts include two which are along the coastline of Ghana and another two inland districts. The Central region has an adult literacy rate of about 50%, with literacy rate among men being higher than that of women (69.8% vs 46.3%). The unemployment rate among men in the region is however slightly lower than that of women (8.0% vs 8.2%) [5].

The baseline survey used clusters listed by the Ghana Statistical Service (GSS) and were used in the Ghana Demographic and Health Survey (DHS). A multistage stratified cluster random sampling process was used to select participants in line with the design for the on-going cluster-Randomised Control trial. Initially, clusters (communities) were randomly selected within each district, after which we randomly selected enumeration areas (EAs) within the selected clusters, and then selected households within the selected EAs using systematic random sampling. We used probability proportional to size (PPS) to select number of EAs within clusters and number of households within EAs. Different EAs were drawn for the men’s survey, separate from those for the women’s survey in each district. A total 10 clusters were selected in each district. An average of 82 households were selected in each of the 10 clusters in each district and an adult male (≥ 18 years) and who is deemed to live (sleep and eat) in the household, and who has lived in the community for at least a year, was invited to participate in the survey. A total of 2126 men were interviewed.

Interviews were conducted in English, Twi or Fante, depending on the participant’s language preference, and data was gathered through face to face interviews and recorded on Personal Digital Assistants. Additional details of the trial design can be found in the study protocol which is registered on Clinical Trials.gov (Identifier: NCTo3237585).

### Measures

#### Physical or sexual intimate partner violence perpetration

The main outcome in this paper is self-reported physical or sexual violence perpetration against an intimate partner in the past 12 months. This dichotomous outcome is derived from 5 physical violence perpetration outcomes and 3 sexual violence perpetration items (Table 1). These were measured on a 4-point scale (1 = none, 2 = once, 3 = few, 4 = many). A participant was deemed to have perpetrated sexual or physical IPV if he had done any of these sexual or physical acts.

**Table 1:** List of items for measuring physical or sexual IPV.

In the last 12 months:
*Physical violence*
• How many times did you slap your current or previous girlfriend/wife, or throw something at her which could hurt her?
• How many times did you push, shove your current or any previous wife/girlfriend?
• How many times did you hit your current or previous girlfriend or wife with a fist or with something else which could hurt her?
• How many times did you kick, drag, beat, choke or burn your current or previous girlfriend or wife?
• How many times did you threaten to use or actually used a gun, knife or other weapon against your current or previous girlfriend/wife?
*Sexual violence*
• How many times have you forced your current or previous girlfriend/wife to have sex with you when she did not want to?
• How many times have you had sex with your current or previous girlfriend/wife when you knew she didn’t want it but you believed she should agree because she is your wife/partner?
• How many times have you forced your current or previous girlfriend/wife to do something sexual that she did not want to do?

Other forms of violence measured were emotional IPV (measured using 4 items), single item economic IPV measure and non-partner violence.

Some of the protective or risk factors, based on previous literature, measured in the baseline survey included childhood experience or exposure to violence in the home, having gender equitable attitudes and relationship practices, being involved in other forms of violence perpetration (fights with other men or violence against non-partners). Other risk factors measured were sexual behaviours, mental health and substance use as well as partner characteristics.

#### Gender attitudes and relationship practices

Gender equitable attitudes were measured using the 8-item Gender Equitable Men (GEM) scale adopted from the WHO Multi-Country Study on Women’s Health and Domestic Violence against Women, and look at the extent to which men agree with separate roles for men and women or agree with equality between men and women [34]. Some of the items in the GEM scale include “A woman’s most important role is to take care of her home and cook for her family” or “A man should have a final word about decisions in his home”. Each item was measured on a 4-point scale (1 = strongly agree, 2 = agree, 3 = disagree, 4 = strongly disagree).

Factor analysis was done to assess the reliability and consistency of the GEM scale in the Ghana context (Cronbach’s alpha coefficient = 0.65) and GEM score was created as an additive scale with higher scores representing more equitable attitudes.

Permissible attitudes towards VAW were measured using two items from the gender attitudes scale. These items were: i) “There are times when a woman deserves to be beaten”, ii) “A woman should tolerate violence in order to keep her family together”. Participants who agreed (agreed or strongly agreed) to either of the two statements, were considered to have permissible attitudes towards VAW.

We also measured individual and community gender norms using 9-item gender relationship scales, adopted from the Stepping Stones/Creating Future study in South Africa [21]. Examples of items measuring societal perceived attitudes towards gender equitability included “My community thinks that a woman should obey her husband”, or “My community thinks that a woman cannot refuse to have sex with her husband”. Similar items, but rephrased in first person, were used to assess individual attitudes. Examples of these were: “I think that a woman should obey her husband”, or “I think that a woman cannot refuse to have sex with her husband”. Additive scores were created for individual and community attitudes after checking for consistency in the item scales (Cronbach’s alpha coefficients were 0.66 and 0.72 respectively), with high scores representing gender equitable norms.

Relationship Control 8-item scale was used to measure the controlling behaviour of the men towards their intimate partners, done with the purpose of exerting power. Items in this scale include: “I won’t let my partner wear certain things”, “I tell my partner who she can spend time with” and “I want to know where my partner is all of the time”. These items were measured on a 4-point Likert scale. The scale had good internal consistency (Cronbach’s alpha coefficient = 0.69). An additive score as a measure of the overall controlling behaviour, with high scores indicating more controlling behaviour.

#### Childhood exposure and experience of violence

Childhood exposure to violence (experienced or witnessed before the age of 18 years) was measured using the Childhood Trauma Scale (CTQ). We used a modified version of the short form of the Childhood Trauma Questionnaire which had a four point Likert scale [35]. However, items were grouped into the following subthemes: i) witnessing abuse of mother by father or boyfriend (1 item), ii) experiencing sexual abuse as a child (3 items), iii) experiencing physical abuse as a child (2 items), iv) experiencing emotional abuse as child (2 items), and v) experiencing parental neglect as a child (4 items). All the items were measured on a 4-point scale (1 = never, 2 = sometimes, 3 = often, 4 = very often). The responses were dichotomised and any participant was deemed to have experienced childhood abuse if they indicated ‘sometimes’, ‘often’ or ‘very often’ to any of the items in the subscales.

#### Mental health and substance use

We used the Centre for Epidemiological Studies Depression Scale (CES_D) to measure level of depression amongst participants [36]. The CES-D is a 20-item scale that measures different aspects of depression such as sleeping problems, feelings of helplessness, eating problems and feelings of guilt among other aspects. We created a score after checking for internal consistency (Cronbach’s alpha coefficient = 0.86). Apart from measuring depression, we also measure Post-Traumatic Stress Disorder (PTSD) using a shortened Harvard Trauma Questionnaire (HTQ) with 16 items (Cronbach’s alpha = 0.90). We asked participants if they had in the past week experienced any of the symptoms. The responses were measured on a 4-point Likert scale: 0 = not at all, 1 = a little, 2 = quite often, and 3 = extremely often. We used the scale guidelines to create a dichotomous variable.

Participants were asked if they had in the course of their life experienced some traumatic events such as ‘witnessing murder of friend or relative’, ‘being victim of armed robbery’ or ‘witnessed someone being raped’. The trauma exposure was measured using an adapted Life Event Checklist from the PTSD checklist (8 items). We created a dichotomous measure from the binary responses and a participant was considered to have experienced or witnessed a traumatic event if they responded positive to at least 1 of the items.

Participants were asked if they use drugs or drink alcohol. On alcohol use, participants indicated how often they take drinks containing alcohol (1 = never, 2 = less than once a month, 3 = 1–3 times a month, 4 = once or twice a week, and 5 = everyday/nearly every day). We then dichotomised the responses based on whether someone ever drinks or not and a combined measure of drug and alcohol use was created.

#### Sexual behaviour and partner characteristics

Sexual behaviour risk factor included having multiple sexual partners and being involved in transactional sex. Participants were asked about the number of main and other partners they had had sex with the past year. Involvement in transactional sex was derived from 5 questions that assessed whether a participant had had sex where the partner expected to get monetary or material support. Partner characteristics that were measured include age of partner, employment status, earning disparity and their education level.

#### Social and demographics

We analysed various social and demographic factors that in previous research have been found to be associated with IPV perpetration such as education level, marital status, employment status, household food security and age of participant [11, 14, 37]. Household food security variable was calculated using the Household Food Insecurity Access Scale (HFIAS) and derived 4-level categorical variable for food security [38]. However, for modelling we combined mildly secure with the moderately insecure categories.

### Ethical issues

Ethics approval for the trial was obtained from the South African Medical Research Council Ethics Committee (EC031-9/2015) and the Institutional Review Board at the Noguchi Memorial Institute for Medical Research at the University of Ghana (# 006/15-16). Eligible participants were given information about the purpose of the study, procedures involved, participants’ rights, risks and benefits of participating in the study in the language of their choice and were enrolled in the survey voluntarily. A researcher was present during the initial informed consent process to ensure that participants understood the information, study procedures and their rights. The informed consent process was done in English, Twi or Fante, depending on the participant’s language preference. Written consent was then sought from all eligible participants prior to commencement of the interview. For safety and confidentiality reasons, only one participant was interviewed in each selected household and interviewers ensured that interviews were done in complete privacy. All participants were given unique identification number and their names were not recorded on the questionnaire. This was done to ensure anonymity of the process. Participants were reimbursed with 10 Ghanaian Cedis (~ 3USD) for their time and inconvenience completing the questionnaire.

### Statistical analysis

In all analysis procedures, we took into account the multistage sampling design of the survey, with stratification by district and the enumeration areas being the lowest level clusters. We assessed whether there was any relationship between non-response to these respective partner characteristic variables and our outcome variable (perpetration of sexual or physical IPV).

Descriptive statistics were presented as mean (with standard deviation) or as frequencies (with percentages). We calculated 95% Confidence Intervals using Taylor linearization. Bivariate relationships between sexual or physical violence perpetration and attitudinal, behavioural and demographic variables were examined using the Pearson’s Chi-Square test for categorical variables and t-tests for continuous variables. We also examined the relationship between childhood experience of violence or exposure to violence and other potential risk factors of IPV perpetration.

To investigate potential risk factors associated with physical or sexual IPV perpetration, we first conducted a bivariate analysis using maximum likelihood logit models. All factors associated with IPV perpetration in the bivariate analysis (p-value<0.05) were put in the multivariate model. We also included any factors which were marginally significant (0.05 <p-value<0.10), which we considered as potential meaningful risk factors. The multivariate logistic regression model adjusted for age of participant. To account for clustering, we used generalised linear mixed modelling, with enumeration areas as random effects.

We also examined the model for multi-collinearity using VIF, and dropped variables that were highly correlated. For example, we found that individual gender attitudes were highly correlated with perceived community gender attitudes (VIF>10). However, individual gender attitude score had a stronger relationship with IPV perpetration than the perceived community gender attitude score. Thus, in final multivariate logistic model we did not include the perceived community attitude score. We further investigated the effect of including perpetration of non-partner violence and perpetration of emotional violence as risk factors.

Of the 2126 participants interviewed, 1973 were married or had a partner/girlfriend in the 12 months preceding the survey. This was the denominator used for our outcome (sexual or physical IPV perpetration in past 12 months). However, all men interviewed had been in some relationship in their lifetime and were all considered for descriptive analysis on lifetime perpetration of IPV. Due to an error in the skip pattern in the questionnaire, respondents that were not living with their partners/girlfriend did not respond to questions on partner characteristics. In order to utilise information on partner characteristics in the modelling, we created a dummy level in all partner characteristic variables (education, age, employment and earning disparity). Apart from partner characteristics, all other variables had very little or no missing data. With very little or no missing information in the explanatory variables, we used listwise deletion in multivariate logistic regression modelling. All analyses were conducted using Stata SE Version 13.

## Results

Table 2 shows the socio-demographics of the participants. The average age of the men in the study was 39.5 years (CI: 38.7–40.4), with about a third (31.5%) of them having reached senior secondary school or above. Overall, 60% of the men were currently married and about 40% of them were experiencing severe household food insecurity. Their average age at first marriage was 26.2 years (CI: 25.8–26.7). Land ownership was at 40% and seven in ten men had earned some money the previous three months. The average number of biological children was less than the average number of children being financially supported (3 children vs 4 children).

**Table 2:** Social and demographic characteristics of the sample of men interviewed in the 4 districts.

CHARACTERISTIC	N (= 2126)‡	% or mean	95% CI§	
			LCL	UCL
**Age of respondent (mean & CI)**	2126	39.5	38.7	40.4
**Education level**				
None	408	19.2	16.5	22.2
Primary	359	16.9	14.7	19.4
Junior Secondary	689	32.4	29.3	35.7
Senior Secondary or above	670	31.5	25.9	37.7
**Current Marital Status**				
Married	1271	59.8	56.4	63.1
separated/divorced/no relationship	322	15.1	13.6	16.9
Not married but in relationship	533	25.1	21.6	28.9
**Food insecurity**				
Secure	692	32.5	28.6	36.7
Mildly/Moderately insecure	576	27.1	24.8	29.6
Severely insecure	858	40.4	36.4	44.5
**Currently Studying**	210	9.9	7.6	12.8
**Club/Society membership**	417	19.6	16.8	22.7
**Active in church/society**	1331	62.6	59.4	65.7
**Moved/travelled for work**	1522	71.6	68.4	74.6
**Work in past year**				
Each Month	897	42.2	39	45.5
Most months	422	19.8	17.5	22.4
Once in a while	500	23.5	20.8	26.5
Never worked	307	14.4	12.6	16.5
**Earned money in previous 3 months**	1295	71.3	68.0	74.3
**Land ownership**	867	40.8	34.2	47.7
**No. of biological children (mean & CI)**	2126	4.1	3.9	4.2
**No. of children financially supporting (mean & CI)**	2126	2.7	2.5	2.9
**Age at first marriage (mean& CI)**	1515	26.2	25.8	26.7

^§:^ Estimation of the Confidence Interval took into account multi-stage design of the study.

^**‡**:^ Total number of men interviewed.

Table 3 shows the prevalence of the different forms of IPV perpetration. Of the 1973 men in intimate relationships in the past 12 months, 235(11.9%) had perpetrated some form of physical violence against a partner, with ‘slapping”, “pushing or shoving” being the most common acts of violence.

**Table 3:** Prevalence of violence perpetration by men.

	PAST 12 MONTHS (N = 1973^¥^)				LIFETIME(N = 2126^‡^)			
	n	%	95% CI^§^		n	%	95% CI^§^	
			LCL	UCL			LCL	UCL
**PHYSICAL IPV ACTS**								
Slapped	121	6.1	4.7	8.0	370	17.4	14.6	20.6
Pushed or shoved	179	9.1	7.3	11.2	408	19.2	16.2	22.6
Hit	75	3.8	2.8	5.1	203	9.5	8.1	11.3
Kicked, dragged, beat, choked or burned	72	3.6	2.7	5.0	207	9.7	7.9	12.0
Threatened with or used weapon	5	0.3	0.1	0.6	12	0.6	0.3	1.0
**Perpetrated any physical IPV**	**235**	**11.9**	**9.7**	**14.6**	**589**	**27.7**	**24.3**	**31.4**
**SEXUAL IPV ACTS**								
Forced partner to have sex when she did not want	245	12.4	10.9	14.2	513	24.1	21.1	27.4
Had sex with partner against her will because you believed she had to.	256	13.0	11.3	14.9	434	20.4	17.7	23.4
Forced to do something sexual	129	6.5	5.4	7.8	158	7.4	6.2	8.9
**Perpetrated any sexual IPV**	**328**	**16.6**	**14.8**	**18.7**	**591**	**27.8**	**25.1**	**30.7**
**Perpetrated any sexual or physical IPV**	**454**	**22.8**	**20.3**	**25.5**	**862**	**40.5**	**37.3**	**43.8**
**OTHER TYPES OF VIOLENCE**								
Economic IPV	121	6.1	4.6	8.1	202	9.5	7.3	12.2
Emotional IPV	455	23.1	20.4	25.9	720	33.9	31.1	36.8
Perpetrated any IPV (including sexual and physical IPV)	697	35.3	32.1	38.7	1096	51.6	48.3	54.8
Perpetrated non-partner violence	195	9.9	8.3	11.7	508	23.9	21.1	26.9

^§:^ Estimation of the Confidence Interval took into account multi-stage design of the study.

^¥:^ Total number of men who had been in a relationship in the 12 months preceding the survey.

^‡:^ Total number of men interviewed and who have ever been in an intimate relationship.

Lifetime perpetration was at 27.7% (589/2126), with ‘slapping”, “pushing or shoving”, also being the most common acts. A total of 328 (16.6%) men had perpetrated some form of sexual violence in past 12 months, with their lifetime prevalence at 27.8%. Overall, a fifth of the men (454/1973) had perpetrated physical or sexual violence against an intimate partner in the 12 months preceding the survey. Almost one- third of the 454 men who had perpetrated sexual or physical IPV, had perpetrated violence against non-partner, and over half (261/454) perpetrated emotional IPV in past year. The prevalence of emotional IPV only was 13%.

Table 4 shows the bivariate relationship between sexual or physical IPV perpetration in the past year and key demographic, behavioural and attitudinal factors. Table 5 shows the unadjusted and adjusted odds ratio for all factors associated with IPV perpetration in bivariate analysis. Risk of IPV perpetration decreased with age (OR = 0.97, CI: 0.97–0.98). There was no significant relationship between IPV perpetration and education level, however men who were in a relationship but not married had higher risk of IPV perpetration (OR = 1.49, CI: 1.08–2.05). IPV perpetration was also highly associated with household food insecurity with men from severe food insecure households being twice (OR = 2.02, CI: 1.53–2.67) likely to perpetrate IPV than those from a food secure household. However, men who had never been employed were less likely to have perpetrated IPV, even after adjusting for age (OR = 0.49, aOR = 0.44).

**Table 4:** Bivariate analysis of factors associated with past year sexual or physical intimate partner violence perpetration.

	N = 1973	No IPV (n = 1523)		IPV (n = 450)	
		n or mean	%/sd	n or mean	%/sd
**Background characteristics**					
Age of participant‡	1973	40.7	15.3	34.5	12.4
Highest Education level					
none	372	302	19.8	70	15.6
primary	334	251	16.5	83	18.4
Junior Secondary	641	480	31.5	161	35.8
Senior Secondary and above	626	490	32.2	136	30.2
Current Marital Status					
Married	1271	1012	66.4	259	56.5
separated/divorced/no relationship	169	125	8.2	44	9.8
Not married but in relationship	533	386	25.3	147	32.7
Household Food insecurity					
Secure	654	547	35.9	107	23.8
Mildly/Moderately insecure	535	414	27.2	121	26.9
Severely insecure	784	562	36.9	222	49.3
Never employed in past year	251	217	14.2	34	7.6
**Childhood Experience Of Violence**					
Witness abuse of mother	358	218	14.3	140	31.1
Experience physical abuse as child	1052	754	49.5	298	66.2
Experienced sexual abuse as child	437	276	18.1	161	35.8
Experienced emotional abuse as child	897	637	41.8	260	57.8
Neglected as child	1269	901	59.2	368	81.8
**Gender attitudes and relation practices**					
Gender attitudes (high = equitable) ‡	1973	17.4	4.8	15.5	4.2
Individual gender norms(high = equitable) ‡	1973	23.4	5.3	20.8	5.1
Controlling behaviour (high = controlling) ‡	1973	21.4	5.0	22.6	4.3
Permissive attitudes towards VAW	1174	834	55.0	340	74.6
**Sexual Behaviour**					
Multiple sexual partners in past 12 months	556	350	23.0	206	45.8
Transactional sex or sex with sex worker	515	311	20.4	204	45.3
** Mental health and substance use**					
Depression score (high = depressed)‡	1973	26.1	8.5	27.7	8.7
PTSD	41	25	1.6	16	3.6
Substance use	884	607	39.9	277	61.6
Experienced traumatic events	704	453	29.7	251	55.8
**Partner Characteristics**¥					
Partner education					
Same	485	407	26.8	79	17.3
respondent more educated	620	484	31.8	136	30.2
Partner more educated	206	139	9.1	69	14.9
N/A	659	490	32.2	169	37.6
Partner unemployed	252	172	11.3	80	17.8
Earning disparity					
Same	144	124		20	4.4
I earn more	965	749	72.6	216	48.0
She earns more	204	159	15.4	45	10.0
N/A	659	490	32.2	169	37.6

^‡:^ Summary statistics represented by mean and standard deviation.

^¥:^ A dummy level (N/A) created for all respondents who did not respond due to an error in the skip pattern in the questionnaire.

**Table 5:** Multivariate logistic regression analysis of the risk factors of past year sexual or physical IPV perpetration.

	Unadjusted		Adjusted	
	OR	95%CI	OR	(95%CI)
**Background characteristics**				
Age of participant‡	0.97	0.96–0.98	0.97	0.96–0.98
Current Marital Status				
Married	Ref	-----	Ref	------
separated/divorced/no relationship	1.38	0.83–2.28	1.08	0.57–2.03
Not married but in relationship	1.49	1.08–2.05	1.03	0.71–1.49
Household Food insecurity				
Secure	Ref	----	Ref	----
Mildly/Moderately insecure	1.49	1.11–2.01	1.28	0.90–1.82
Severely insecure	2.02	1.53–2.67	1.25	0.90–1.72
Never employed in past year	0.49	0.31–0.78	0.62	0.38–1.02
**Childhood Experience Of Violence**				
Witness abuse of mother	2.70	2.06–3.54	1.40	1.07–1.88
Experience physical abuse as child	2.00	1.64–2.43	1.10	0.80–1.51
Experienced sexual abuse as child	2.52	1.94–3.26	1.61	1.21–2.15
Experienced emotional abuse as child	1.90	1.49–2.43	1.29	0.97–1.73
Neglected as child	3.10	2.37–4.05	1.80	1.31–2.47
**Gender attitudes and relation practices**				
Gender attitudes (high = equitable) ‡	0.91	0.88–0.94	1.00	0.96–1.04
Individual gender norms (high = equitable) ‡	0.91	0.88–0.93	0.94	0.91–0.97
Controlling behaviour (high = controlling) ‡	1.06	1.02–1.09	1.01	0.97–1.04
Permissive attitudes towards VAW	2.48	1.91–3.21	1.21	0.89–1.63
**Sexual Behaviour**				
Multiple sexual partners in past 12 months	2.83	2.13–3.75	1.71	1.23–2.38
Transactional sex or sex with sex worker	3.23	2.58–4.05	1.77	1.37–2.28
** Mental health and substance use**				
Depression score (high = depressed)‡	1.02	1.01–1.03	1.00	0.98–1.02
Substance use	2.37	1.74–3.22	1.83	1.30–2.56
Experienced traumatic events	2.98	2.38–3.73	2.07	1.65–2.60
**Partner Characteristics**				
Partner education				
Same	Ref	-----	Ref	--------
respondent more educated	1.02	0.77–1.36	1.36	0.99–1.89
Partner more educated	1.75	1.24–2.47	1.40	0.88–2.28
Partner unemployed	1.70	1.30–2.22	1.60	1.15–2.22

^‡:^ continuous measures

Experience of any form of childhood abuse was associated with IPV perpetration, with men who had experienced physical neglect being three times more likely to perpetrate IPV (OR = 3.10, CI: 2.37–4.05), and men who had experienced sexual abuse were two times more likely to perpetrate IPV (OR = 2.52, CI: 1.94–3.26). Similar risks were observed in those that had experienced physical abuse (OR = 2.00, CI: 1.64–2.43) or emotional abuse (OR = 1.90, CI: 1.49–2.43). Witnessing abuse of mother was also strongly associated with IPV perpetration (OR = 2.70, CI: 2.06–3.54).

We also found very significant association between experience of violence or exposure to violence in childhood and having attitudes that endorse VAW. Men who had witnessed abuse of their mother were three times more likely to have attitudes that endorse VAW (OR = 3.3 CI: 2.5–4.3). There were significant associations between permissible attitudes towards VAW and childhood neglect or physical abuse (OR = 1.7 and OR = 1.3 respectively). Childhood experience or exposure to violence was also highly associated with substance use. Men who had witnessed abuse of their mother were two times more likely to use alcohol or drugs.

Men who perpetrated sexual or physical IPV were more likely to perpetrate sexual violence against a non-partner (OR = 11.2, CI: 7.8–16.0), and were more likely to perpetrate emotional violence against their intimate partner (OR = 9.04, CI: 6.98–11.9).

Having risky sexual behaviour was also highly associated with IPV perpetration. Men with multiple partners in the past 12 months were three times more likely to have perpetrated sexual or physical IPV (OR = 2.83, CI:2.13–3.75). Similarly, men who had been involved in transactional sex or had had sex with sex workers were three times more likely to have perpetrated sexual or physical IPV (OR = 3.23 CI: 2.58–4.05).

Use of alcohol or drugs was also associated with IPV perpetration, with men who drink or use drugs being twice more likely to perpetrate IPV (OR = 2.41 CI: 1.94–3.00). Risk of IPV perpetration increased with increase in depression score (OR = 1.02, CI: 1.01–1.03). Having experienced some traumatic events was also highly associated IPV perpetration (OR = 2.98 CI: 2.38–3.73). However, having post-traumatic stress disorder was partially associated with IPV perpetration (p-value = 0.07). Substance use was highly associated with having multiple partners (OR = 1.7 CI: 1.3–2.1), being involved in transactional sex (OR = 2.4 CI: 1.9–3.1) and having experienced/witnessed some traumatic events (OR = 1.6 CI: 1.3–1.9).

Having attitudes that condone VAW was also a strong risk factor for IPV perpetration. Men with permissible attitudes towards VAW were two times more likely to perpetrate IPV (OR = 2.48, CI: 1.91–3.21). Having gender equitable attitudes reduced a man’s likelihood of perpetrating IPV (OR = 0.91 CI: 0.88–0.94). However, there were no significant differences in what the respondents indicated were community gender norms between perpetrators and non-perpetrators. Controlling behaviour was also found to be significantly associated with IPV perpetration, with the odds of IPV perpetration increasing with increase in controlling behaviour (OR = 1.06 CI:1.02–1.09).

Of the men who provided information about their partner, partner unemployment and partner education were most significant factors associated with IPV perpetration. Men with partners who were more educated than them were more likely to perpetrate IPV compared to men who had the same education levels with their partners (OR = 1.75 CI: 1.24–2.47). Most of the men (181/206) with more educated partners had junior secondary school level or lower. A man with an unemployed partner was two times more likely to perpetrate IPV than a man whose partner was employed (OR = 1.70 CI:1.30–2.22).

In the multivariate logistic regression (Table 5), childhood experience of violence (sexual and neglect) and exposure to violence as a child (witnessing abuse of mother) are some of the factors that remained highly associated with IPV perpetration. Risky sexual behaviours (multiple partners and transactional sex) were also significantly associated with IPV perpetration. Other risk factors were substance use, experiencing/witnessing some traumatic events, having individual gender inequitable attitudes and having an unemployed partner. Severe food insecurity, being unemployed or education level disparity were not significantly associated with IPV perpetration in the multivariate model.

## Discussion

Our findings show high levels of sexual or physical IPV perpetration in the selected communities in Ghana with one in five men having perpetrated sexual or physical IPV in the 12 months preceding the survey, and one in ten men having perpetrated physical IPV in 12 months preceding the survey. Sexual IPV prevalence was higher than physical IPV prevalence, and not all perpetrators used all types of violence. However, there was some overlap between sexual and physical IPV, with 6% of the men perpetrating both sexual and physical IPV in the 12 months preceding the survey. Findings from the UN Multi-country study showed variation in the occurrence of sexual or physical IPV with some countries having higher prevalence of sexual IPV than physical IPV and other countries having higher physical IPV prevalence compared to sexual IPV [14]. There are suggestions from literature that the higher prevalence of sexual IPV perpetration relative to physical IPV could be due to prevalent social attitudes, norms and practices (cultural or religious) that allow men to exert control and force over women [9, 14]. In Ghana, status of women is determined by traditional norms and practices as well as religious values that promote the subordination of women. Based on evidence from a qualitative study, Gadzeko urges that men’s control over women is driven by men’s belief that women are a possession and may be linked to the dowry system, which is practiced across the country. Men believe they are entitled to demand sex from the partner whether the woman wants it or not [39]. The lower estimates for physical IPV perpetration could be due to men being less willing to disclose physical assault against intimate partner.

For lifetime prevalence rates, almost one in every three men had perpetrated some form of physical IPV. Our lifetime prevalence rates are far much higher than findings by Kishor and Bradley, who using data from nationally representative Demographic and Health Surveys in Ghana found a lifetime physical IPV perpetration of 16.3% [40]. However, this Demographic and Health Survey data did not have measures for sexual IPV perpetration. Generally, there is very limited data on men’s IPV perpetration in Ghana and in most sub-Saharan countries, with most studies looking at women’s experience of IPV. Thus, the importance of this study in providing evidence for IPV perpetration and its associated risk factors in Ghana cannot be overemphasised.

In this paper, we found that having witnessed abuse of one’s mother increases the risk of IPV perpetration in adulthood. The very significant association between witnessing inter-parental violence and having attitudes that endorse VAW bridges the link between exposure to violence in childhood and IPV perpetration later in life. This is consistent with other studies that have looked at inter-generational transmission of VAW [11, 41–43]. The increased likelihood of IPV perpetration among men who witnessed the abuse of their mother is driven in part by psychosocial concepts from the Social Learning Theory that have shown that individuals learn how to behave through observing and imitating important individuals in their social environment [44]. The impact of social learning and cognitive learning is more pronounced in children and the youth. We also found that men who had witnessed abuse of their mother were more likely to use drugs or drink alcohol. This finding reflects other studies which have also shown significant association between witnessing inter-parental violence and drug or alcohol abuse [45, 46]. Consistent with other studies, we found a strong association between experiencing neglect as a child and IPV perpetration [43, 45, 47, 48]. Furthermore, we found that men who had ever experienced or witnessed traumatic events had high likelihood of perpetrating IPV. Witnessing or experiencing traumatic events was also strongly associated with substance use. This is congruent with other studies that have shown linkage between witnessing/experiencing traumatic events, substance/alcohol misuse and violence perpetration [18, 19, 49, 50].

In the crude bivariate analysis, both gender attitude measure from the GEM scale and individual attitudes score were strongly associated with IPV perpetration and perceived community gender attitudes was marginally significant. In the multivariate analysis, only individual attitudes were significantly associated with IPV perpetration. However, there was a very strong positive correlation between GEM score and individual gender attitudes score, which might explain the non-significant relationship between GEM score and IPV perpetration in the multivariate model. Our findings are in line with several studies that have found a relationship between gender attitudes and IPV perpetration or experience [3, 14, 27]. Despite men with attitudes that condone VAW having high likelihood of perpetration in the bivariate analysis, the effect was not significant when adjusted for other factors. However, several studies have highlighted permissible attitudes towards VAW as one of the risk factors of IPV perpetration across different settings [29, 31, 51]. It is of note therefore that we found that it was individual gender attitudes that were important in predicting violence perpetration and not just permissive attitudes towards it. Furthermore, individual attitudes were more important than perceived community gender attitudes. These findings do not support some of the assertions currently being made in the intimate partner violence field which are emphasising community level social norms and attitudes to violence over individual level attitudes [25]. There are important implications for interventions.

Field and colleagues found alcohol consumption was a more influential predictor of IPV perpetration than permissible attitudes towards violence [52]. Apart from alcohol being used as an excuse for misbehaviour and risk taking, it can also reduce inhibitions, and/or cloud one’s judgement [4, 18, 49]. Studies have also linked alcohol use with aggressive behaviour, in that men are more likely to act violently when drunk [4, 53].

Bivariate analysis further showed a strong relationship between IPV perpetration and woman’s education level relative to their partner’s education level, with men whose partners were more educated than them having high likelihood of perpetrating IPV. In a study done in India, Ackerson and colleagues found increased likelihood of IPV experience among women with no education but also in women who were more educated than their partner [54].

Findings from the Ghana 2008 DHS showed that women with tertiary education had decreased likelihood of experiencing IPV[55]. However, Ghana DHS did not examine the effect of a woman’s education relative to her spouse’s education level. High education attainment increases a woman’s chances of gainful employment, which may in turn result in wealth. Jewkes highlights the U-shaped relationship between IPV perpetration/experience and a woman’s education attainment [4]. Education attainment levels in our study population like most rural communities, was generally low, with about a third of the men having senior secondary school level or above. Of these men, less than 10% had more educated partners. Despite women’s education attainment being protective against IPV experience, the female partners in this study did not have high enough levels of education to benefit from the education effect. The higher risk of IPV perpetration amongst men with more educated partners may be perpetuated by the imbalance partner’s education causes to the traditional gender roles and hierarchy’. The risk may also emanate from the men’s gender inequitable attitudes as they are likely to believe they should be the ones providing for their women and families [17]. Education has also been found to be associated with permissive attitudes towards VAW, with more highly educated population having less prevalent attitudes that condone violence against women.

Our study found strong association between risky sexual behaviours (having multiple partners and engaging in transactional sex) and IPV perpetration, a finding consistent with that of other studies that have looked at risk factors for IPV perpetration/experience and generally interpreted these as indicator variables for a more patriarchal, sexually entitled masculinity [13, 14, 17, 28]. Furthermore this finding supports other studies that have linked abusive male partners with increased risk of STI/HIV among women [8, 17, 28, 56, 57].

Having an unemployed partner increased men’s likelihood of perpetrating IPV. Several studies have shown that female empowerment through education or income is generally protective against IPV [11, 13]. High levels of women empowerment help to bridge the power imbalance between men and women. Food insecurity was to be a risk factor for IPV perpetration in the bivariate analysis, which is consistent with findings from the UN-Multi-Country study [14]. Despite the association not being highly significant in the multivariate analysis, men from food insecure households had higher odds of IPV perpetration than those from food secure households. There is a growing body of knowledge that supports the co-occurrence of psychosocial problems (food insecurity, depression problem drinking), risky sexual behaviour (multiple sexual partners, involvement in transactional sex) and IPV perpetration [18, 20, 26, 28, 49, 58, 59].

A major limitation of our study is the non-response to partner’s characteristics. Information on partner characteristics would have helped in linking IPV perpetration with women’s characteristics relative to men’s. Another limitation of our study is the unavailability of data at community level that could have helped us understand society-level drivers of IPV perpetration. The findings of this study cannot to be generalised to the whole of Ghana, but provide much needed empirical evidence on which primary VAW prevention interventions can be based, and act as a reference level for the Rural Response System (RRS) intervention to reduce gender-based violence currently ongoing in the districts where the survey was conducted. Based on the cross-sectional study design, we are also mindful that it is not possible to infer causal relationships between IPV perpetration and the risk factors examined in our paper.

## Conclusions

The study contributes important evidence about factors associated with men’s IPV perpetration in Ghana, and complements findings from studies conducted amongst women. Findings of this study emphasise the urgent need for primary prevention interventions that can address inter-generational transmission of violence and address learned gender inequitable attitudes and norms that condone men’s control and dominance over women. Interrupting the cycle of violence is very critical in reducing men’s perpetration of VAW. Context-specific evidence is critical for designing appropriate and relevant interventions and policies. We also argue that it may be necessary for VAW primary prevention interventions to take into account contextual challenges like poverty and unemployment [60].

## Supporting information

S1 File This file contains data collected from 2126 participants. (CSV)
